# Human *PTCHD3 *nulls: rare copy number and sequence variants suggest a non-essential gene

**DOI:** 10.1186/1471-2350-12-45

**Published:** 2011-03-26

**Authors:** Mohammad M Ghahramani Seno, Benjamin YM Kwan, Ka Ki M Lee-Ng, Rainald Moessner, Anath C Lionel, Christian R Marshall, Stephen W Scherer

**Affiliations:** 1 The Centre for Applied Genomics and Program in Genetics and Genome Biology, The Hospital for Sick Children, Toronto, Ontario M5G 1L7, Canada; 2 Department of Pathobiology, School of Veterinary Medicine, Shiraz University, Shiraz, Iran; 3 McLaughlin Centre and Department of Molecular Genetics, University of Toronto, Toronto, Ontario M5S 1A8, Canada

## Abstract

**Background:**

Copy number variations (CNVs) can contribute to variable degrees of fitness and/or disease predisposition. Recent studies show that at least 1% of any given genome is copy number variable when compared to the human reference sequence assembly. Homozygous deletions (or CNV nulls) that are found in the normal population are of particular interest because they may serve to define non-essential genes in human biology.

**Results:**

In a genomic screen investigating CNV in Autism Spectrum Disorders (ASDs) we detected a heterozygous deletion on chromosome 10p12.1, spanning the Patched-domain containing 3 (*PTCHD3*) gene, at a frequency of ~1.4% (6/427). This finding seemed interesting, given recent discoveries on the role of another Patched-domain containing gene (*PTCHD1*) in ASD. Screening of another 177 ASD probands yielded two additional heterozygous deletions bringing the frequency to 1.3% (8/604). The deletion was found at a frequency of ~0.73% (27/3,695) in combined control population from North America and Northern Europe predominately of European ancestry. Screening of the human genome diversity panel (HGDP-CEPH) covering worldwide populations yielded deletions in 7/1,043 unrelated individuals and those detected were confined to individuals of European/Mediterranean/Middle Eastern ancestry. Breakpoint mapping yielded an identical 102,624 bp deletion in all cases and controls tested, suggesting a common ancestral event. Interestingly, this CNV occurs at a break of synteny between humans and mouse. Considering all data, however, no significant association of these rare *PTCHD3 *deletions with ASD was observed. Notwithstanding, our RNA expression studies detected *PTCHD3 *in several tissues, and a novel shorter isoform for *PTCHD3 *was characterized. Expression in transfected COS-7 cells showed *PTCHD3 *isoforms colocalize with calnexin in the endoplasmic reticulum. The presence of a patched (Ptc) domain suggested a role for *PTCHD3 *in various biological processes mediated through the Hedgehog (Hh) signaling pathway. However, further investigation yielded one individual harboring a homozygous deletion (*PTCHD3 *null) without ASD or any other overt abnormal phenotype. Exon sequencing of *PTCHD3 *in other individuals with deletions revealed compound point mutations also resulting in a null state.

**Conclusion:**

Our data suggests that *PTCHD3 *may be a non-essential gene in some humans and characterization of this novel CNV at 10p12.1 will facilitate population and disease studies.

## Background

Unbalanced submicroscopic genetic variation, known as copy number variation (or CNV), is now well studied [1-4]. CNV can potentially contribute to variable degrees of fitness and/or disease predisposition [5-7]. In a recent high-resolution microarray study of CNV in humans, it was determined that at least 0.78% of the genome is CNV between any two individuals [4]. Analyses of the sequence assembly of a diploid genome at higher resolution determined that approximately 1.28% was CNV [8,9]. While CNVs, and in particular CNV deletions, are more often observed in gene poor regions [3,4], in the highest resolution population study to date, 3,811 of the validated biallelic deletions detected across 41 healthy individuals were found to overlap 1,432 genes [4].

Homozygous CNV deletions, herein called CNV nulls, affecting genes have previously been reported in the human genome [10]. Though loss of gene functions mostly reduce fitness, under certain conditions complete loss of functions can be beneficial [11,12]. For example, it is reported that homozygous mutations affecting *DARC *or *CCR5 *result in resistance to certain categories of malaria [13] or AIDS [14,15], respectively, without posing any overt deleterious effect on the individuals carrying these mutations.

Here, we discover and characterize a CNV null region in the human genome encompassing the *PTCHD3 *gene that does not appear to be associated with an overt phenotype. Breakpoint mapping indicated a recurrent 102,624 bp deletion, suggesting a single ancestral event that is now present in ~0.6-1.6% of individuals of European ancestry. Expression analyses showed *PTCHD3 *in several tissues with the highest levels detected in lymph node, testes and tongue. A previously uncharacterized shorter isoform was also found and PTCHD3 isoforms were determined to co-localize with calnexin in the endoplasmic reticulum in transfected cells.

## Methods

### Sample Sets

The Ethics Board of Hospital for Sick Children approved this study and all individuals providing samples signed informed consents covering aspects of the experiments conducted. Population control samples originated from other studies and they were consented and collected precisely for the aforesaid purpose (manuscripts citing the source of the population control samples are mentioned below).

Initial screening of *PTCHD3 *deletions used an index sample set of 427 probands diagnosed with ASD, all of which met the criteria by the Autism Diagnostic Interview-Revised (ADI-R) and Autism Diagnostic Observation Schedule (ADOS) [16]. We also screened another 177 cases from the same ASD population for the deletion using qPCR. Additional control cohort microarray data include 1,152 individuals from Ontario of predominately (95%) European ancestry genotyped on Affymetrix 500 K arrays [17], 1,123 Northern Europeans from the German PopGen project [18] genotyped on the Affymetrix 6.0 SNP array, and 1,234 individuals of European decent from the Ottawa River Valley [19] genotyped on the Affymetrix 6.0 SNP array. DNA panels used for quantitative PCR screening include 1,043 individuals from the HGDP-CEPH Human Genome Diversity Panel [20], and a panel of 186 North American Caucasians (Coriell Institute, USA). Ancestry of deletion carriers was either self reported or in the cases where microarray results were available, through inference using the SNP genotypes clustered with the HapMap samples as described previously [16].

### Affymetrix GeneChip Human Mapping 500 K SNP and 6.0 Microarrays and CNV analysis

Affymetrix 500 K experiments and CNV analysis for the ASD cases and Ontario controls were carried out as described previously [16]. CNV analysis of Affymetrix 6.0 arrays is described elsewhere [21].

### PTCHD3 Deletion Validation and Population Screening

For all cases where DNA was available, putative *PTCHD3 *deletions were validated with SYBR-Green I-based real time quantitative PCR (qPCR) using the *CFTR *locus as a reference as described previously [16]. The same assay was used to screen the 177 additional ASD probands, the 186 from the North American Caucasian Panel, and the 1,043 individuals from the HGDP-CEPH for *PTCHD3 *deletions.

### Deletion Breakpoint Analysis and Exon Sequencing

Putative *PTCHD3 *deletions in ASD and HGDP-CEPH individuals were amplified with Stratagene Taq2000 polymerase and sequenced for breakpoint analysis. PCRs product sizes of either 3 kb (primers PCR01F and PCR01R) or 2 kb (primers PCR01F and SEQ03R) were used for sequencing using one or more of the primers SEQ01F, SEQ01R, SEQ02R, SEQ03R, and SEQ04R (see Additional file 1 for primer sequences). Sanger dideoxy-DNA sequencing and Exon-sequencing of the PTCHD3 (NM_001034842) in deletion carrier families was carried out using 12 different amplicons. Primer sequences and PCR conditions used for amplification are available on request.

### Antibodies

Mouse monoclonal anti-Myc (sc-40); rabbit polyclonal anti-calnexin (sc-11397) and goat anti-mouse HRP-conjugated secondary antibody (sc-2005) were purchased from Santa Cruz biotechnology (USA). Alexa Fluor 488 goat antimouse IgG1 (A-21121) and Alexafluor 555 goat anti-rabbit IgG (A-21429) secondary antibodies were purchased from Invitrogen.

### PTCHD3 Cloning and Transfection

Using the primer set C (Additional file 2) the full-length isoform of *PTCHD3 *was amplified from a pCR-Bunt II-TOPO plasmid containing the full length human *PTCHD3*. Primer set C was designed so that the amplicons would have *BamH1 *and *Xba1 *restriction sites at their 5' and 3' ends, respectively. For the shorter isoform of *PTCHD3*, cDNA from human lymph node was initially amplified using the primer set D designed based on sequences at/or around 5'UTR and 3'UTR regions of *PTCHD3*, and the ~2,000 bp product of this amplification was then used as a PCR template for primer set C. The ~1,600 bp amplified product of the latter then was used for cloning and further characterisation. The amplified long and short isoforms were then cloned into TOPO A vectors (Invitrogen) for further amplification in Top 10 competent cells (Invitrogen) following the instruction provided by the manufacturer. Finally, the long and short isoforms were both cloned into the *BamH I*-*Xba I *site of a pcDNA3 vector (Invitrogen) which had already been cloned with a c-Myc expressing motif at its *Kpn I*-*BamH I *sites. COS-7 cells were grown in DMEM medium containing 10% FBS. For western blot assay, the cells were transfected with the plasmids expressing *PTCHD3 *using Genejuice transfection reagent (Novagen) according to the instructions provided by the manufacturer. 48 hours later the cells were lysed in western blot lysis buffer and transferred to -20°C until used for further analysis. For immunocytochemistry, COS-7 cells were grown in chamber slides (Lab-Tek, Miles Laboratories) and transfected with the plasmids using Genejuice as explained above. 48 hrs later cells were used for immunolabelling as described below.

### Western Blotting

Cell lysates were loaded on a 12% Polyacryamide gel and the resolved proteins were transferred to a nitrocellulose membrane following the general protocol for western blotting. The membranes were blocked in 5% milk for 1 hour and then were incubated with the 1:1,000 dilution of anti-Myc primary antibody in blocking buffer (5% milk) for another hour. After three washes of 10 minutes each, the membrane was incubated with 1:4,000 dilution of anti-mouse HRP-conjugated secondary antibody for 45 minutes. Membranes were washed three times, 10 minutes each and were developed using western blotting chemiluminescence reagents (PerkinElmer).

### Immunocytochemistry

Cells were briefly washed with PBS and fixed in 4% formaldehyde at room temperature for 20 minutes. The fixed cells were permeablized in 0.1% triton (Sigma) in PBS at room temperature for 10 minutes, washed twice with PBS and were blocked in 10% Bovine Serum Albumin (BSA) (Sigma) in PBS at room temperature for 1 hour. The cells were then incubated in 1:100 dilution of anti-Myc and anti-calnexin primary antibodies in 3% BSA at room temperature for 1 hour. After 3 washes of 3 minutes each in PBS, the cells were incubated with 1 μg/ml of each of Alexa Fluor 488 goat antimouse IgG1 and Alexafluor 555 goat anti-rabbit IgG in 3% BSA for 45 minutes. After three washes in PBS, cells were studied using confocal microscopy.

### Multiple Tissue Northern (MTN) Blot

The probe was amplified using primer set shown in Additional file 2, and the cDNA prepared from human lymph nodes. Probe labelling was conducted using ^32^P-labelled dCTP (PerkinElmer) following the protocol explained below. 1.2 μl of 0.1 U/μl random hexamers mix (GE Healthcare) was added to 7.2 μl of DNA probe (11 ng/μl), boiled in a water bath for 2 minutes and placed immediately on ice. 10 μl of 2.5× random priming buffer (0.5 M HEPES pH 6.6, 12.5 mM MgCl_2_, 28.8 mM β-mercaptoethanol, 125 mMTris pH 8.0 and 0.05 mM dATP/dGTP/dTTP mix) and 1 μl of 10× BSA (New England Biolabs Inc.) were added to the probe reaction and incubated at 22°C for 10 minutes followed by addition of 4 U of DNA Poly I Klenow fragment (USB corp.) and 5 μl (1.85 MBq) of [α^32^P]dCTP. The reaction was incubated at 22°C for 4 hours. 25 μl of TE with 0.1% SDS was added to the labelled probe and the unincorporated nucleotides were removed by centrifugation at 700 g for 2 minutes on a ProbeQuant G-50 Sephadex micro column (GE Healthcare). The flow through (labelled probe) was collected and the specific activity was measured using a QC-2000 reader (BioScan). The labelled probe was then boiled for 10 minutes and added to 5 ml of warm (68°C) buffer (clontech). The probe mixture was then added to a Multiple Tissue Northen blot membrane (Clonetch) that had been prehybridized in ExpressHyb buffer for 1 hour at 68°C. The hybridization proceeded at 68°C for 16 hours. The membrane was washed and exposed overnight against Biomax XAR autoradiograph film (Kodak) at -80°C and developed.

## Results and Discussion

### Discovery and Population Distribution of PTCHD3 Deletion

Using Affymetrix 500K arrays to investigate structural variation in individuals with Autism Spectrum Disorder (ASD), we detected a recurrent heterozygous deletion at chromosome 10p12.1. From our initial assessment the deletion was found at a frequency of ~1.4% (6/427) and was observed to intersect a single annotated gene, Patched-domain containing 3 (*PTCHD3*). In all ASD cases the deletion was found to be inherited, but nonetheless was determined to be an interesting candidate gene since it had not previously been described in control populations. Additionally, we and others recently described deletions in the X-linked *PTCHD1*, a *PTCHD3 *homologue, to be associated with ASD and intellectual disability [16,21-23]. We then tested an additional 177 ASD probands and found two more heterozygous deletions, bringing the total to 1.3% (8/604) (Table 1). All probands were of European ancestry. To determine the frequency in population controls, we initially used a qPCR assay to screen a panel of North American Caucasians and found the heterozygous deletion at a similar frequency of ~1.6% (3/186). Subsequent analysis of Affymetrix array data in three populations of predominately European ancestry yielded heterozygous *PTCHD3 *deletion frequencies of ~0.67% (8/1,152) [17], 0.36% (4/1,123) [18], and 0.97% (12/1,234) [19] (Table 1). Though the deletions were at slightly elevated frequency in ASD, we did not see a statistically significant association over controls when considering all data (p = 0.13; Fisher's 2-tailed exact test) (Table 1).

**Table 1:** PTCHD3 deletions frequencies in Autism and control populations

Population	Origin	Method	Total Number unrelated	PTCHD3 del count	Frequency (%)	Reference
Autism	Canada	500 K array	427	6	1.40	Marshall [16]
	
	Canada	qPCR	177	2	1.13	unpublished

***Total***			***604***	***8***	***1.32***	

Control	Canada	500 K array	1,152	8	0.69	Zogopolous [17]

	Other	qPCR	1,043	7	0.67	HGDP

	N.America	qPCR	186	3	1.61	Coriell, USA

	Germany	Affy6.0	1,123	4	0.36	Krawczak [18]

	Canada	Affy6.0	1,234	12	0.97	Stewart [19]

***Total***			***4,552***	***31***	***0.68***	

p-value for cases versus controls is not significant (Fisher's extact two tail; P = 0.13). 8/604 and 31/3695 + 648 = 4343 excluding the African, East Asian, and Oceania populations from the HDGP samples.

**Figure 1:**
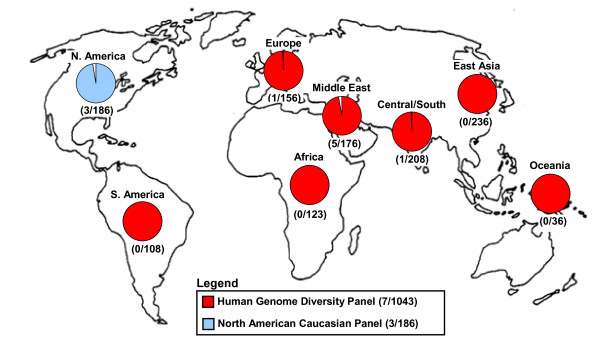
**Geographical population distribution of *PTCHD3 *deletions**.

Although no obvious role for involvement in ASD was found, we sought to determine the population frequency and possible ancestral origin of the deletion by characterizing the human genome diversity panel (HGDP-CEPH), which is comprised of DNA samples from worldwide populations [20]. We found heterozygous *PTCHD3 *deletions in ~0.67% (7/1,043) individuals in the HGDP-CEPH (Figure 1) including three of Palestinian descent (from Israel Central), one of Druze descent (from Israel Carmel), one of Balochi descent (from Pakistan) and one of Northern Italian descent (from Italy Bergamo). Thus the deletion appeared to be present in those of Mediterranean/Middle East descent, suggesting this population as the likely origin of the ancestral CNV event. When we had sufficient family histories on the autism families to assess geneology, they were also found to have links to the Mediterranean/Middle East regions.

### Breakpoint Characterization

**Figure 2:**
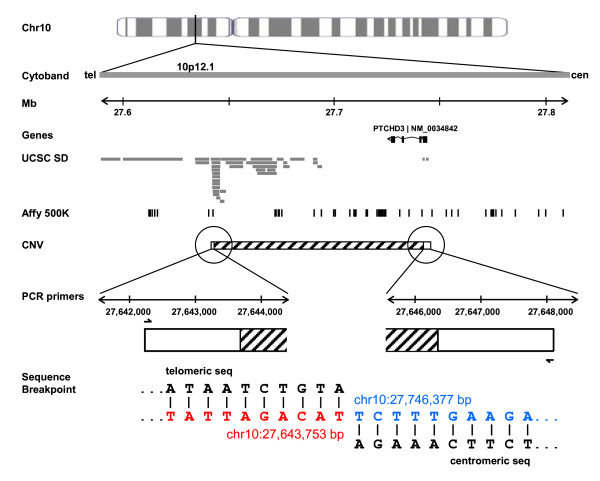
**Genomic characterization of the *PTCHD3 *deletion variant**. The cytoband and genomic coordinates (NCBI Build 35) are expanded from the chromosome 10 ideogram. The *PTCHD3 *gene (black), UCSC segmental duplications (grey bars), and Affymetrix 500 K SNP probes (black lines) are also shown. The deletion is denoted by a rectangle with hatched bars showing the sequenced breakpoints. Expansion of the region shows the location of primers (arrows) used for sequencing with the telomeric (red) and centromeric (blue) breakpoint sequences.

Using several PCR assays (Figure 2, Additional file 1) we defined and sequenced the breakpoints of the deletion, eventually determining it was 102,624 base pairs in size (spanning Chr.10: 27,643,753-Chr.10:27,746,377; NCBI Build 35). The breakpoint was found to be identical in the eight ASD probands and seven HGDP-CEPH individuals tested, suggesting a single ancestral event. The distal 10p12.1 breakpoint resides in a segmental duplication while the proximal end does not. Interestingly this *PTCHD3 *CNV region corresponds to a human-murine break of synteny with portions of murine chromosome 2 (inverted), chromosome 11 (inverted) and chromosome 18 mapping to human 10p12.1 [24] (Additional file 3).

### No Obvious Clinical Phenotype in *PTCHD3 *Nulls

**Figure 3:**
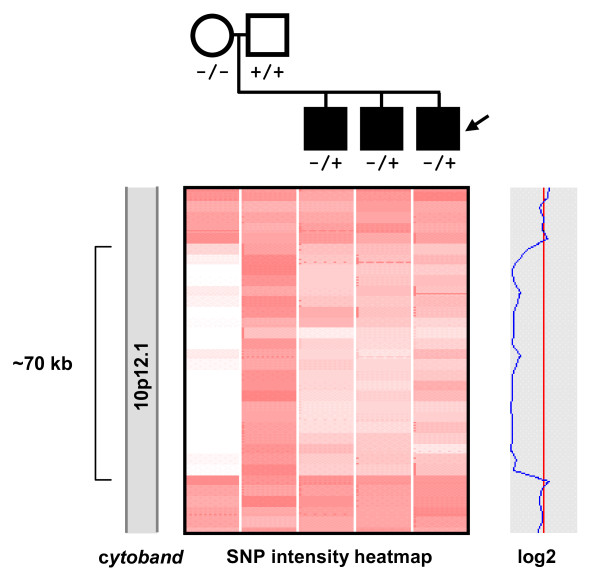
**Segregation of a homozygous *PTCHD3 *deletion**. The '-' denotes the deleted allele and '+' the non-deleted wild type allele. Females are denoted by circles and males with squares. Individuals with a diagnosis of ASD are black with the proband denoted by an arrow. The heatmap profiles below show individual probe intensities covering the deleted region (top, telomeric end) with white, pink, and red signifying copy numbers of 0, 1, and 2, respectively. The blue line shows the smoothed log2 ratio for the mother.

Of the eight ASD probands with deletions, we were able to determine maternal inheritance in four families (SK0191, SK0136, SK0257, MM0303) and paternal inheritance in two families (SK0145, MM0145). For two of the families, we did not have parental DNA. In one family, SK0191, all three offspring were found to carry the deletion and the mother was found to be homozygous for the deletion. We confirmed transmission by running the entire family (mother, father, proband, two affected siblings) on the Affymetrix 500 K array and subsequent PCR validation and breakpoint determination (Figure 3). The mother with the homozygous deletion has no apparent abnormal phenotype.

To further characterize *PTCHD3*, we sequenced exons in the families harboring deletions (Additional file 4). We found two novel sequence variants that, in combination with the deletions, result in a null genotype. The father in family MM0145 has a deletion (CNV) on one allele compounded with a single base pair deletion at A1767. This single nucleotide deletion results in a frameshift introducing a stop codon at ~46 bp downstream of the deleted base suggesting no functional *PTCHD3 *copy in this individual. However, this nucleotide variant only affects the larger *PTCHD3 *isoform as the sequence harboring it is excluded from the novel shorter isoform we identified (see below). Family members from both SK0145 (father) and SK0136 (male proband) (Additional file 4) have CNV deletions and single base pair insertions at 923G. Again, this single nucleotide insertion, which in this case affects both the long and short *PTCHD3 *isoforms, results in a frameshift introducing a stop codon at ~75 bp downstream of the inserted base. Both fathers have no obvious clinical phenotype and have fathered children despite the suggested role of *PTCHD3 *in sperm motility [25], indicating *PTCHD3 *is not essential for fertility.

### PTCHD3 Expression Profile and Functional Analysis

**Figure 4:**
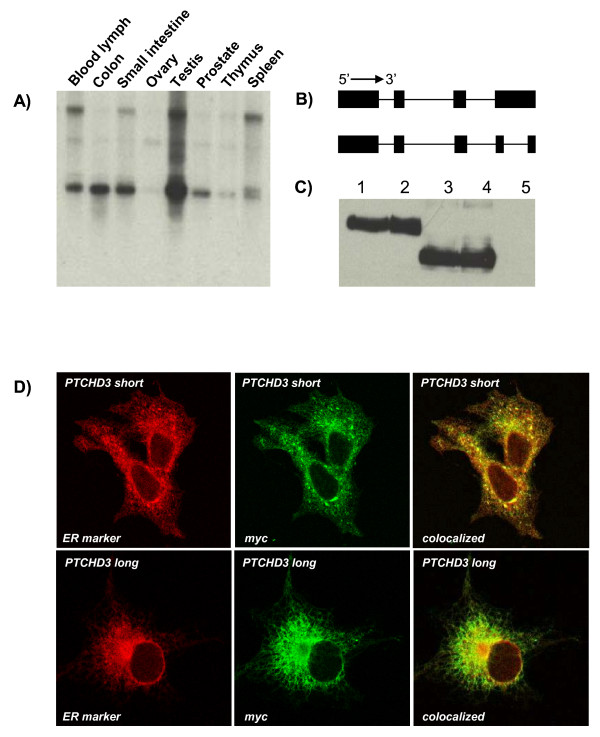
**Expression and localization of *PTCHD3 *isoforms**. A: A multiple tissue northern blot demonstrating the expression profile of *PTCHD3 *isoforms in different tissues. B: A schematic demonstration of two predominant isoforms of *PTCHD3 *showing truncation of the last exon of previously documented *PTCHD3 *isoform (coding sequence: 2,304 bps) in the newly identified isoform (coding sequence: 1,602 bps). C: Cloning and expression of *PTCHD3 *isoforms tagged with a Myc epitope in COS-7 cells resulted in detection (by western blot) of two proteins of ~115 KDa (lanes 1 and 2) and ~80 KDa (lanes 3 and 4); lane 5: run with the lysates prepared from untransfected cells. D: Both *PTCHD3 *isoforms are localised to the endoplasmic reticulum (ER). Immunostaining of COS-7 cells transfected with constructs expressing Myc-tagged short or longer isoforms of *PTCHD3 *using antibodies detecting calnexin (red) as an ER marker and Myc epitope (green) demonstrated co-localization of *PTCHD3 *isoforms with calnexin to the ER (Pearson coefficient 0.85).

Due to the lack of an obvious phenotype in *PTCHD3 *nulls, we sought to further characterize the gene and rule out the possibility of *PTCHD3 *being a pseudogene. Using primer set A (Additional file 2) *PTCHD3 *expression was evaluated by PCR in cDNA panels of adult and fetal tissues. Although mouse *Ptchd3 *expression is testis-specific [25], the RT-PCR performed on a diverse panel of human cDNAs showed widespread expression in adult tissues (pancreas, placenta, salivary gland, skin, spleen, thymus, thyroid, trachea, bone marrow, brain, colon, heart, kidney, lung, lymph node, tongue, testis, ovary, spinal cord) and foetal sources (brain, bladder, kidney, lung, spleen and stomach) (data not shown). We did not detect expression of *PTCHD3 *in adult mammary gland, skeletal muscle, stomach, adrenal gland, cerebellum, fibroblasts, liver, uterus and foetal liver, skeletal muscle, thymus and aorta (data not shown). Expression appeared to be highest in adult lymph node, testes and tongue. Using a probe detecting both *PTCHD3 *isoforms, the *PTCHD3 *expression was further confirmed by northern blot on RNAs from 8 human tissues (Figure 4A). Despite apparent null genotypes in some individuals, *PTCHD3 *is expressed in human tissues.

Using primer set B (Additional file 2), expression in lymph nodes showed amplification of a long and a short isoform of *PTCHD3 *running on an agarose gel at ~2.3 kb and ~1.6 kb, respectively (Additional file 5). Both isoforms were cloned into and expressed from a pcDNA3 myc vector for further functional characterization. Plasmids containing either short (1,602 bp from, and including, start to stop codons) or long (2,304 bp from, and including, start to stop codons) *PTCHD3 *isoforms were transfected into COS-7 cell line resulting in the expression of a ~80 or ~115 kDa proteins, respectively, as determined by Western blotting (Figure 4C). Immunocytochemistry analysis using anti-Myc antibodies demonstrated co-localization of both of the *PTCHD3 *isoforms with calnexin to the endoplasmic reticulum (Pearson correlation coefficient 0.85) (Figure 4D). The sequence of the newly characterized *PTCHD3 *isoform has been submitted to the GenBank (Genbank accession number: JF332167).

Two *Ptchd3 *isoforms have been detected in mouse, *Ptchd3a *(AK017136) (coding for 410 amino acids) and *Ptchd3b *(AB235902) (coding for 906 amino acids), of which the first encoded 409 amino acids are identical [25]. In mice, *Ptchd3 *expression is developmentally regulated and detected exclusively in testes [25]. Human PTCHD3 shares ~63% amino acids identity with its mouse orthologue and it has been shown that in both species PTCHD3 is expressed and localized to the midpiece of sperm, suggesting possible involvement of PTCHD3 in sperm motility and hence fertility [25]. However, we report healthy offspring fathered from males in which both germline *PTCHD3 *alleles are apparently inactive. It is possible that PTCHD3 can improve fecundity, but its absence may not always be accompanied by infertility.

The hedgehog (Hh) signaling pathway (including Hh protein and its receptor Ptc) has important roles in embryonal patterning and development in both vertebrates and invertebrates [26]. PTCHD3 has Ptc and Sterol Sensing (SSD) domains suggesting a possible role for PTCHD3 in hedgehog signaling. Although, we cannot exclude the possibility of *PTCHD3 *having an important function in humans, our observation indicate that the absence of *PTCHD3 *has no overt effect on normal development. Nonetheless, whether there are some other molecules compensating for *PTCHD3 *loss, or whether this molecule might have beneficial effects under certain conditions remains to be determined. It is noteworthy that different individuals with the same heterozygous deletions, e.g. 16p11.2 microdeletions [27-30], can present a spectrum of phenotypes. In another example, a sib pair was recently reported to both have large homozygous chromosomal deletion (812-902 kb) at chromosome 12q21.1, but while one sib demonstrated dysmorphic features and developmental delay, the other child was unaffected [31]. The variable penetrance may arise due to unshared genes between the sibs, highlighting the need to interpret CNV data in a genome-wide context [7,32].

## Conclusion

We identified a novel 102.6 kb CNV ancestral deletion on chromosome 10p12.1 that is predominantly present in European/Middle Eastern populations and encompasses the *PTCHD3 *gene. A novel shorter ~1.6 kb isoform of *PTCHD3 *(Genbank accession number: JF332167) was also characterized. Expression studies revealed that both the long and short *PTCHD3 *isoforms co-localize with calnexin to the endoplasmic reticulum. We demonstrate that a *PTCHD3 *null state can exist in humans through homozygous deletions, or combinations of deletions and single nucleotide mutations, with no overt abnormal phenotype being associated. Our data helps further define the essential human gene set.

## List of Abbreviations

CNV: Copy Number Variation; ASD: Autism Spectrum Disorders; MTN: Multiple Tissue Northern Blot; Hh: Hedghog; HGDP: Human Genome Diversity Panel; BSA: Bovine Serum Albumin.

## Competing interests

The authors declare that they have no competing interests.

## Authors' contributions

MMGHS, BK, KKML, RM, ACL, and CRM performed laboratory and computational analyses. MMGHS, RM, CRM and SWS designed the study, interpreted the data and wrote the manuscript. All authors read and approved the manuscript.

## Pre-publication history

The pre-publication history for this paper can be accessed here:

http://www.biomedcentral.com/1471-2350/12/45/prepub

## Supplementary Material

Additional file 1 **A Table listing the primers used for breakpoint mapping**. Click here for file

Additional file 2 **A table listing primers used for RT-PCR, cloning and northern blot**. Click here for file

Additional file 3 **A figure depicting murine break of synteny at *ptchd3 *region versus human**. Click here for file

Additional file 4 **Pedigrees demonstrating compounded *PTCHD3 *mutations complexion in families with individuals affected with ASD**. Click here for file

Additional file 5 **A figure depicting *PTCHD3 *expression in human lymph node (L.N.) detected by RT-PCR**. Click here for file
