# Supplementary material for: Human PTCHD3 nulls: rare copy number and sequence variants suggest a non-essential gene
Source: BMC Med Genet. 2011 Mar 26;12:45. doi: 10.1186/1471-2350-12-45 (PMC3072306; doi:10.1186/1471-2350-12-45)
Supplement: Additional file 1 — A Table listing the primers used for breakpoint mapping. [file 1471-2350-12-45-S1.PDF]

Additional file 1. Primer sequences used for breakpoint mapping.

**Primer Name**	**Primer Sequence**	**Use**
PCR01F	GGTAAGCCATAGGTGAGCAATAA	PCR Fragment
PCR01R	CTTTGATGATGGTGACATACAGAT	PCR Fragment
SEQ01R	GCCAAACCAAATGAGATGCT	Sequencing
SEQ02R	CCCAAGGTGCTGGGATTAC	Sequencing
SEQ03R	AGAGGCCAGGTTTCACCATATT	Sequencing, PCR Fragment
SEQ01F	TCATGGTAAGTTATCCTTTGAGTGG	Sequencing
SEQ04R	CAAGGTGCTGGGATTACAGG	Sequencing
