# Supplementary material for: Human PTCHD3 nulls: rare copy number and sequence variants suggest a non-essential gene
Source: BMC Med Genet. 2011 Mar 26;12:45. doi: 10.1186/1471-2350-12-45 (PMC3072306; doi:10.1186/1471-2350-12-45)
Supplement: Additional file 2 — A table listing primers used for RT-PCR, cloning and northern blot. [file 1471-2350-12-45-S2.PDF]

Additional file 2. Primer sequences used for RT-PCR, Cloning and Northern blot.

Primer Set	Primer Seq.-Forward	Primer Seq.-Reverse
A	ACCGGCTTCCTGTACCTACC	TGCCAAGAAAGCAGAAATCA
B	AATCTCAGGAATCGGAGTCG	TGGCTGAGGAAGAAACATCTG
C	CGGGATCCATGCCGTGGGTGG	GCTCTAGATTAGAATAACAATATTTTCTTT
D	TTTCACCTGCTGTTCCTCCT	AGCAAAAGTCACCCACAAGG
Northern blot	GAGTCGGAAGGCAAGCAG	CTAGGCAGTCGGTGTGACAG
