# Supplementary material for: Human PTCHD3 nulls: rare copy number and sequence variants suggest a non-essential gene
Source: BMC Med Genet. 2011 Mar 26;12:45. doi: 10.1186/1471-2350-12-45 (PMC3072306; doi:10.1186/1471-2350-12-45)
Supplement: Additional file 3 — A figure depicting murine break of synteny at ptchd3 region versus human. [file 1471-2350-12-45-S3.PPT]

## Slide 1
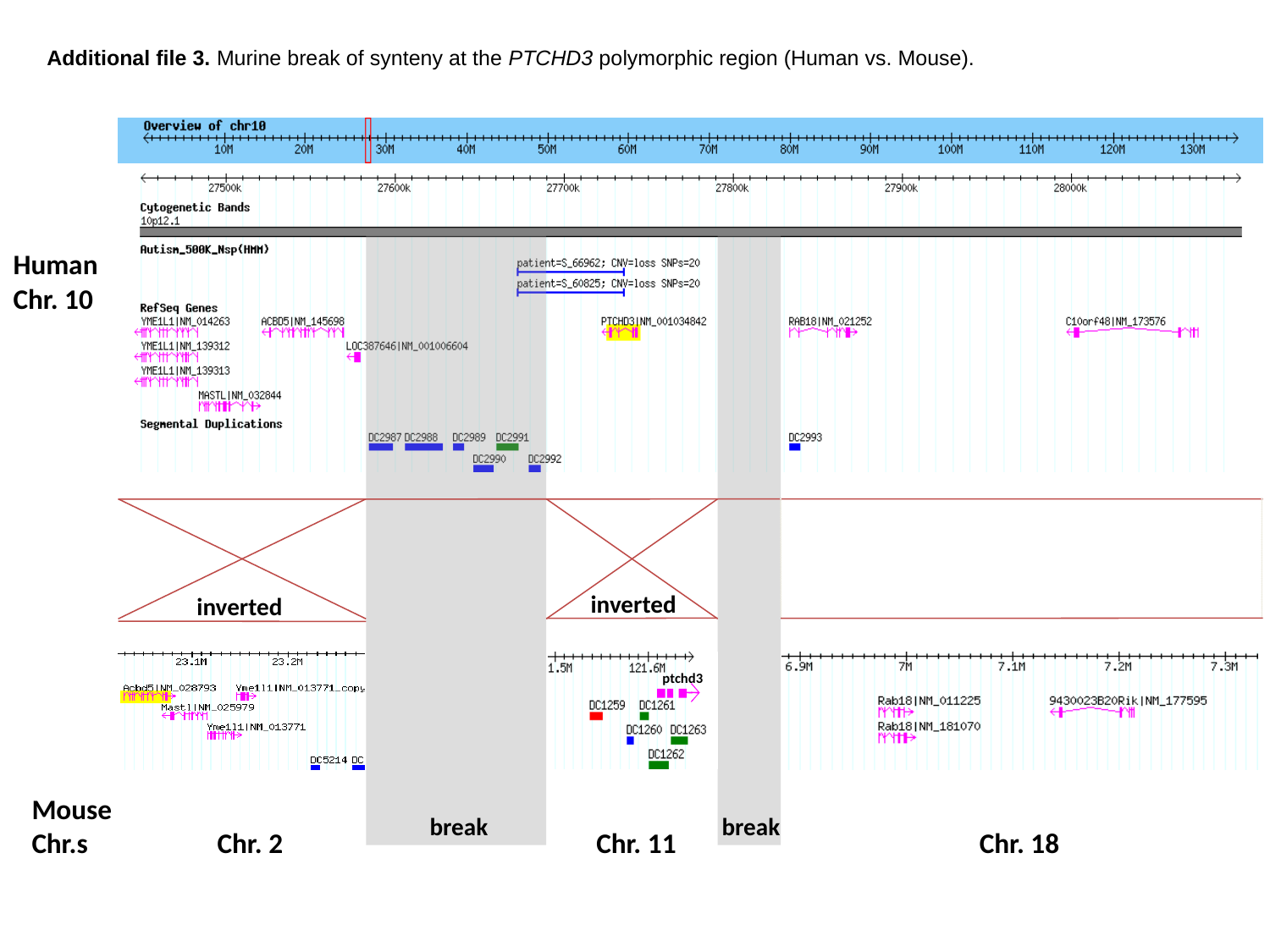

Additional file 3. Murine break of synteny at the PTCHD3 polymorphic region (Human vs. Mouse).
Human
Chr. 10
inverted
inverted
ptchd3
Mouse
Chr.s
break
break
Chr. 2
Chr. 11
Chr. 18
