# Supplementary material for: Human PTCHD3 nulls: rare copy number and sequence variants suggest a non-essential gene
Source: BMC Med Genet. 2011 Mar 26;12:45. doi: 10.1186/1471-2350-12-45 (PMC3072306; doi:10.1186/1471-2350-12-45)

Additional file 4. Compounded *PTCHD3* mutations in families with individuals affected with ASD. The pedigrees demonstrate single nucleotide mutations and CNVs resulting in *PTCHD3* deletion in four families with individuals affected with ASD. Del: deletion, A: Adenine, G: Guanine. The numbers by the bases show the genomic position from start codon.

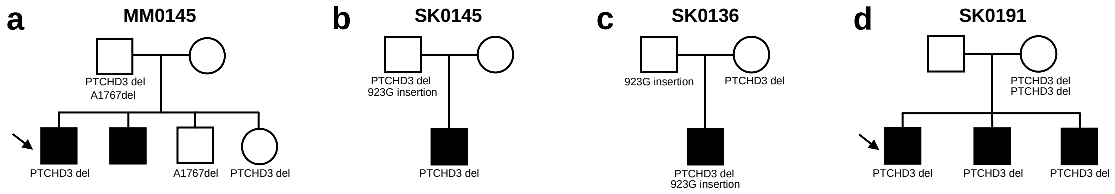

Supplement: Additional file 4 — Pedigrees demonstrating compounded PTCHD3 mutations complexion in families with individuals affected with ASD. [file 1471-2350-12-45-S4.PDF]
