# Supplementary material for: Human PTCHD3 nulls: rare copy number and sequence variants suggest a non-essential gene
Source: BMC Med Genet. 2011 Mar 26;12:45. doi: 10.1186/1471-2350-12-45 (PMC3072306; doi:10.1186/1471-2350-12-45)

Additional file 5. *PTCHD3* expression in human lymph node (L.N.) detected by RT-PCR. Primer set B (see Materials and Methods) was used for this experiment. A long and a short isoform for PTCHD3 are detected.

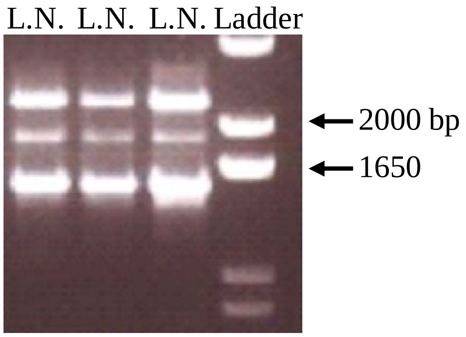

Supplement: Additional file 5 — A figure depicting PTCHD3 expression in human lymph node (L.N.) detected by RT-PCR. [file 1471-2350-12-45-S5.PDF]
